# Interferon-α/β and Anti-Fibroblast Growth Factor Receptor 1 Monoclonal Antibody Suppress Hepatic Cancer Cells In Vitro and In Vivo

**DOI:** 10.1371/journal.pone.0019618

**Published:** 2011-05-09

**Authors:** Shigeru Sasaki, Tadao Ishida, Minoru Toyota, Akinobu Ota, Hiromu Suzuki, Akinori Takaoka, Hiroshi Yasui, Hiroyuki Yamamoto, Hideyasu Takagi, Masahiro Maeda, Tsutomu Seito, Masayuki Tsujisaki, Yasuhisa Shinomura, Kohzoh Imai

**Affiliations:** 1 First Department of Internal Medicine, Sapporo Medical University, Sapporo, Japan; 2 Department of Molecular Biology, Sapporo Medical University, Sapporo, Japan; 3 Immuno-Biological Laboratories Co., Ltd., Fujioka, Japan; 4 Division of Signaling in Cancer and Immunology, Institute for Genetic Medicine, Hokkaido University, Sapporo, Japan; 5 Department of Gastroenterology and Hematology, Tenshi Hospital, Sapporo, Japan; 6 Division of Novel Therapy for Cancer, The Advanced Clinical Research Center, The Institute of Medical Science, The University of Tokyo, Tokyo, Japan; Yonsei University College of Medicine, Republic of Korea

## Abstract

**Background:**

Hepatocellular carcinoma (HCC) is the most commonly occurring primary liver cancer and ranks as the fifth most frequently occurring cancer, overall, and the third leading cause of cancer deaths, worldwide. At present, effective therapeutic options available for HCC are limited; consequently, the prognosis for these patients is poor. Our aim in the present study was to identify a novel target for antibody therapy against HCC.

**Methodology/Principal Findings:**

We used Western blot and flow cytometric and immunocytochemical analyses to investigate the regulation of FGFR1 expression by interferon-α/β in several human hepatic cancer cell lines. In addition, we tested the efficacy of combined treatment with anti-FGFR1 monoclonal antibody and interferon-α/β in a murine xenograft model of human HCC. We found that interferon-α/β induces expression of FGFR1 in human HCC cell lines, and that an anti-FGFR1 monoclonal antibody (mAb) targeting of the induced FGFR1 can effectively inhibit growth and survival of HCC cells *in vitro* and *in vivo*. Moreover, the combination of interferon-α, anti-FGFR1 mAb and peripheral blood mononuclear cells (PBMCs) exerted a significant antitumor effect *in vitro*.

**Conclusions:**

Our results suggest that the combined use of an anti-FGFR1 antibody and interferon-α/β is a promising approach to the treatment of HCC.

## Introduction

Hepatocellular carcinoma (HCC) is the most commonly occurring primary liver cancer and ranks as the fifth most frequently occurring cancer, overall, and the third leading cause of cancer deaths, worldwide [1]. At present, surgery, percutaneous therapies such as ethanol injection and radiofrequency ablation, and transcatheter therapies such as arterial chemoembolization are employed in the treatment of HCC. These approaches can selectively remove and kill cancer cells, which makes them useful for control of the local tumor; however, they are not sufficient to improve the prognosis of HCC patients, as the disease readily recurs due to blood-born metastases (e.g., intrahepatic metastasis and vascular infiltration) or the development of new HCCs (multicentric carcinogenesis). Consequently, the 1-year and 3-year survival rates for HCC are only 36% and 17%, respectively [2]. The weaknesses of the current HCC treatments include incomplete inhibition of multicentric carcinogenesis, difficulties in controlling intraportal infiltration, and the inability to prevent deterioration of hepatic functional reserve or foster its restoration. Thus development of new treatments that improve the prognosis of HCC patients and which can also be used in elderly and advanced stage patients would be highly desirable.

Targeting cell surface molecules using mAbs is an emerging strategy in cancer therapy, and mAbs against cancer-related surface molecules such as EGFR, HER2 and CD20 have been successfully employed [3], [4], [5]. However, cell surface expression of antigenic molecules is often weak and heterogeneous, which prevents the efficient targeting of tumors [6] and, to date, only a few pilot studies examining expression of HCC-associated antigens have been carried out [7].

Interferons (IFNs), which are widely used for the treatment of neoplasias and viral diseases, enhance expression of several cell surface molecules both *in vitro* and in xenograft tumor models [8], [9]. Induction of gene expression by IFN is a complex phenomenon that involves activation of target genes via phosphorylation of STATs by JAK kinase [10]. In addition, IFNs can induce expression of interferon regulatory factors (IRFs) and transcription factors, which then induce genes involved in apoptosis and immune responses [11]. IFNs are already being used to treat most hepatitis patients, and their effects suggest targeting cell surface molecules induced by IFN may be a useful strategy for treating HCC. Our aim in the present study was to use HCC cell lines and a murine xenograft model of human HCC to examine the changes in gene expression induced by IFN and to identify potential targets for antibody therapy. Our findings suggest IFN-α/β-induced fibroblast growth factor receptor 1 (FGFR1) could be a novel therapeutic target for the treatment of HCC.

## Results

### Induction of FGFR1 expression by IFN-α/β in HCC xenografts

**Figure 1:**
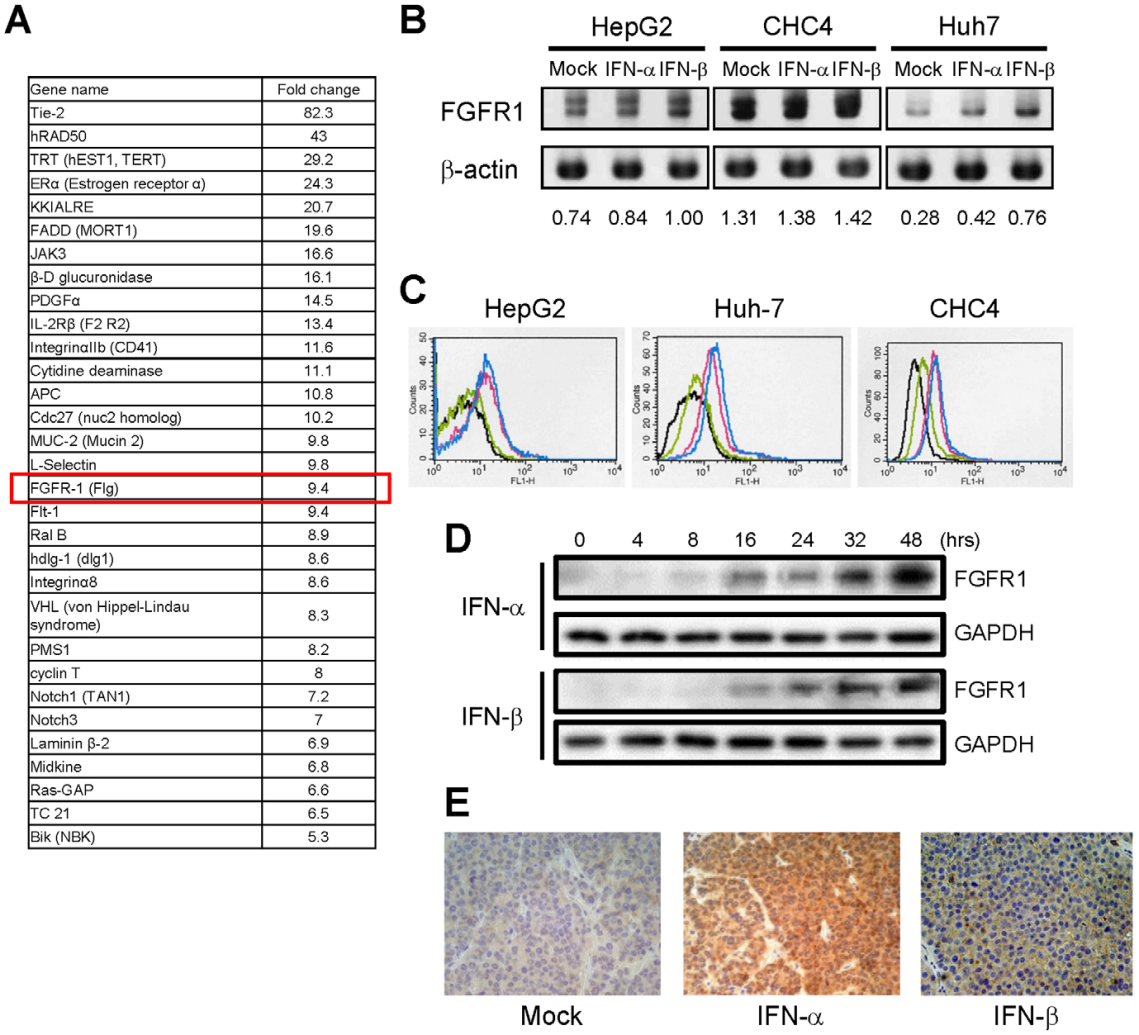
Induction of FGFR1 by IFN-α/β treatment in hepatic cancer cells. A, Summary of genes induced by IFN-α in hepatic cancer cell lines and HepG2-xenografts. Expression of genes induced by IFN-α was examined using DNA array analysis, and expression of *FGFR1* was found to be strongly induced. B, Western blot showing IFN-α/β-induced expression of FGFR1 in human hepatic cancer cells. Relative protein levels are indicated below. C, Flow cytometric analysis showing IFN-α/β-induced expression of FGFR1 in human hepatic cancer cells: black, no antibody; green, no IFN; Pink, IFN-α; Blue, IFN-β. D, Western blot showing the time course of FGFR1 expression after treatment with IFN-α/β. The immunoblots were done using total lysates from HepG2-xenografts. E, IFN-α/β-induced FGFR1 expression in excised tissues from HepG2-xenografts. FGFR1 was stained using anti-FGFR1 antibody.

To identify genes up-regulated by IFN in HCC cells, we performed a microarray analysis using cDNA prepared from tumors grown in SCID mice subcutaneously administrated HepG2 cells, a human hepatic cancer cell line. The results of the microarray analysis are summarized in Figure 1A. Among the genes up-regulated by IFN was *FGFR1*, which encodes a receptor tyrosine kinase. Real-time PCR analysis confirmed induction of *FGFR1* transcription by both IFN-α and IFN-β (Figure S1), and corresponding increases in FGFR1 protein were observed in HepG2, Huh-7 and CHC4 cells (Figure 1B–D). We then used immunohistochemical staining to examine the distribution of IFN-α/β-induced FGFR1 within the tumors and found that levels of FGFR1 were increased at the cell membrane and in the cytoplasm of HCC cells (Figure 1E).

### Development of an anti-FGFR1 monoclonal antibody

**Figure 2:**
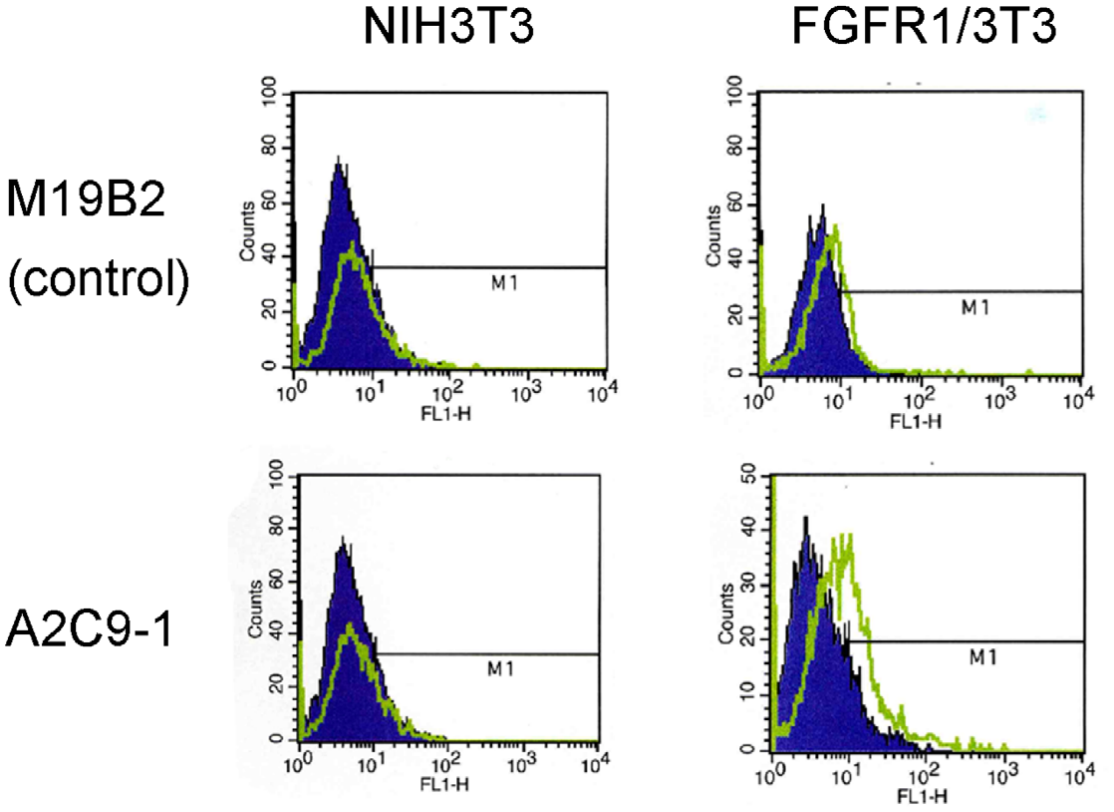
Development of anti-FGFR1 mAbs. Flow cytometric analysis showing the expression level of FGFR1 and specificity of A2C9-1 mAb. Left: the graphs show the results obtained with lysates from NIH3T3 cells into which M19B2 cDNA introduced (control). Right: the graphs show the results with lysates from NIH3T3 cells into which full-length FGFR1 cDNA was introduced.

We developed novel anti-FGFR1 mAbs by immunizing BALB/c mice with an FGFR1 expression vector. Six antibodies recognizing FGFR1 were isolated from the mice, two of which, designated A2C9-1 and A2D11-1, showed strong affinity in ELISAs and were characterized further. For kinetic analyses, the extracellular domain of FGFR1 was covalently coupled to a CM-5 sensor chip at low density (215 response units of FGFR1), after which we determined the Kd values for A2C9-1 and A2D11-1 to be 209 nM and 7.03 µM, respectively (Figure S2A). Thus A2C9-1 showed the strongest affinity for FGFR1. Flow cytometric analysis confirmed that A2C9-1 reacts with FGFR1 (Figure 2), and Western blot analysis showed the molecular weight of the ectopically expressed FGFR1 to be around 115 kDa (Figure S2B).

### Anti-FGFR1 mAbs inhibit HCC cell growth in vitro

**Figure 3:**
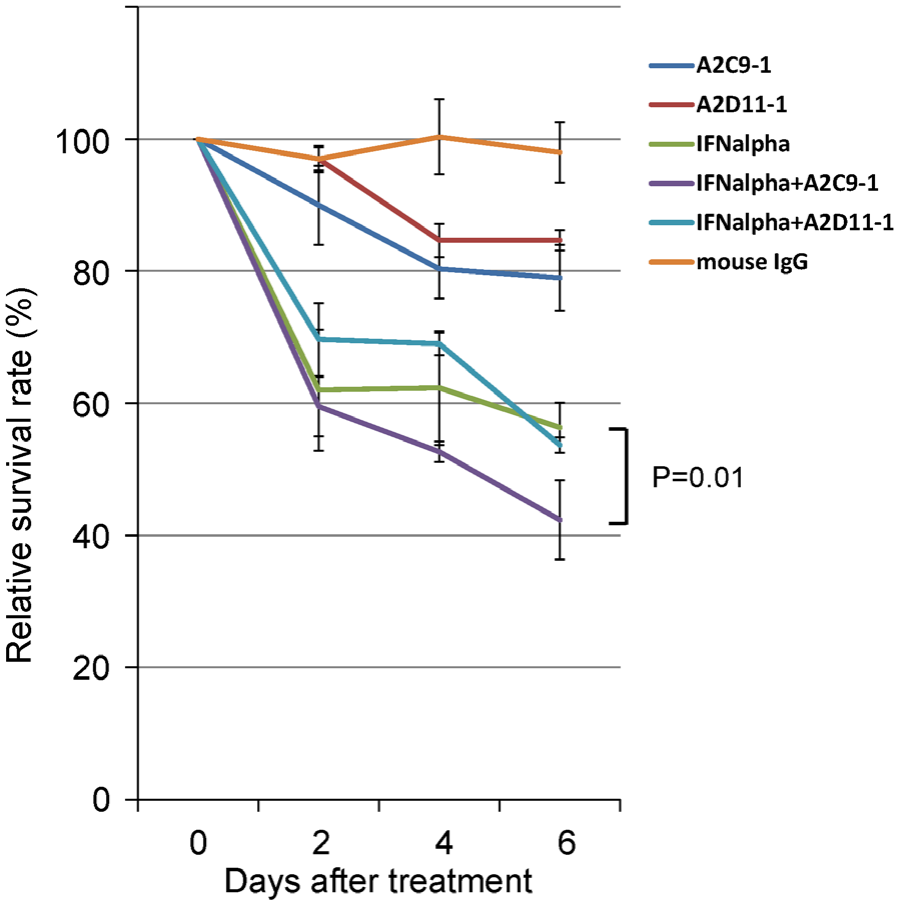
Antitumor activity of anti-FGFR1 mAbs in combination with IFN-α *in vitro.* In the graph, the survival rate among cultured HepG2 HCC cells is shown on the vertical axis, and the incubation time after administration of the indicated drugs is shown on the horizontal axis. PBS is the negative control. Symbols and bars represent means ± SD. Note that treatment with a combination of A2C9-1 and IFN-α significantly reduced cell survival, as compared to treatment with IFN-α alone (*P* = 0.01).

We next examined the effects of A2C9-1 and A2D11-1 mAbs on the growth of hepatic cancer cells (Figure 3). IFN-α showed some antitumor activity against hepatic cancer cells, and weak growth suppression was seen when A2C9-1 or A2D11-1 was added to cultures in the absence of IFN-α. On the other hand, treatment with a combination of A2C9-1 and IFN-α significantly reduced cell survival, as compared to treatment with IFN-α alone (*P* = 0.01) (Figure 3). The effect of A2D11-1 in combination with IFN-α was no greater than the effect of IFN-α alone.

### Effects of A2C9-1 with and without IFN-α in a mouse xenograft tumor model

**Figure 4:**
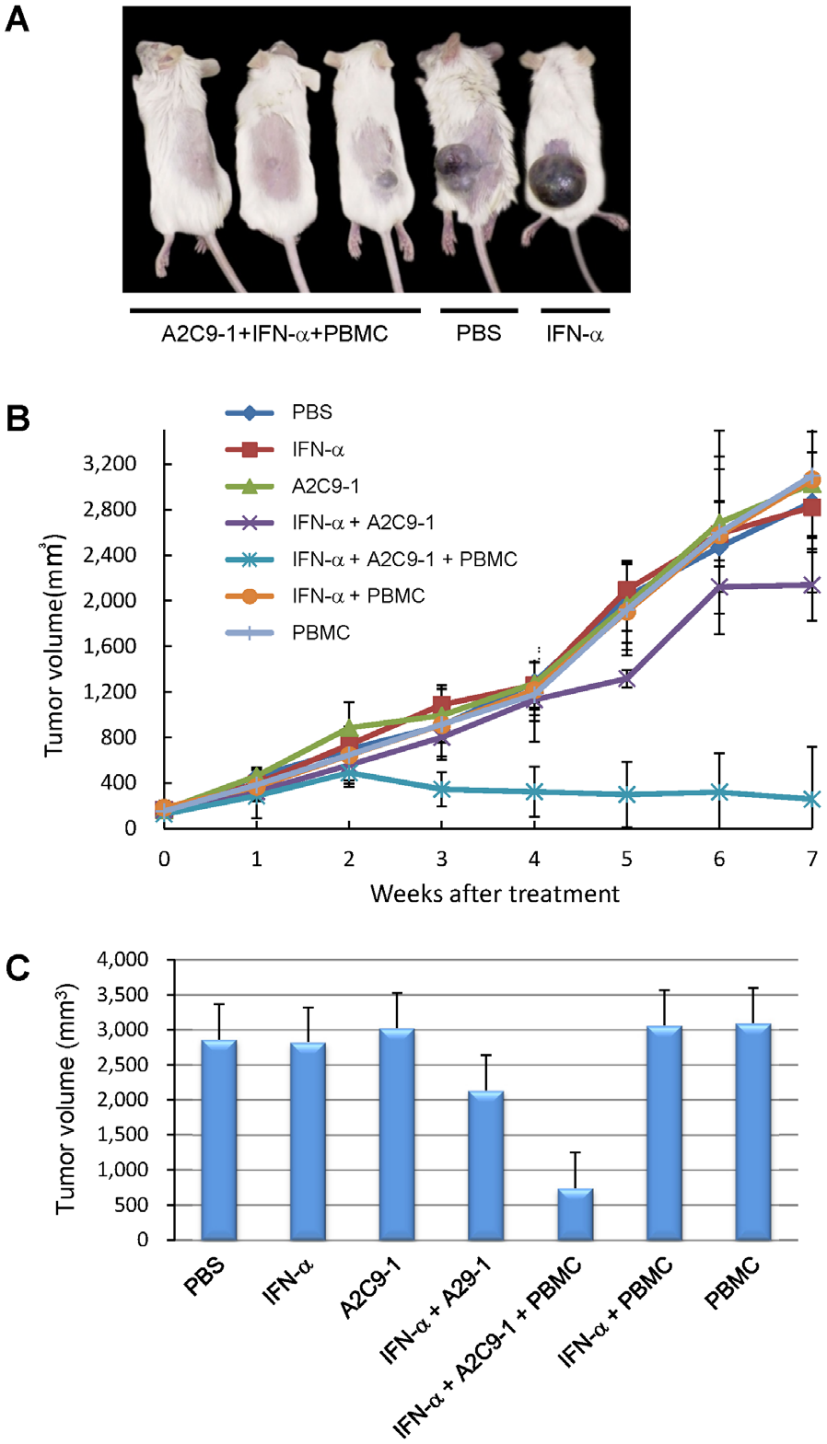
Antitumor activity of anti-FGFR1 mAb in combination with IFN-α in a murine xenograft tumor model of human HCC. A, Representative photos of tumor grafts on SCID mice. B, In the graph, the sizes of the tumors in each group (n = 4 mice per group) are shown on the vertical axis, and the elapsed time after treatment with the indicated drugs and cells is shown on the horizontal axis. Symbols and bars are means ± SD. C, Tumor volumes following treatment with the indicated drugs and cells. PBS is the negative control. Data are means ± SD.

We next tested the antitumor effects of an anti-FGFR1 mAb in a mouse xenograft model of human HCC (Figure 4A and B). In mice treated with only A2C9-1 or IFN-α, tumor volumes did not differ from the control group administered PBS. However, treatment with IFN-α+A2C9-1 had an inhibitory effect on tumor growth, though the suppression was not statistically significant. Finally, in mice treated with IFN-α+A2C9-1+PBMCs (peripheral blood mononuclear cells), there was a significant antitumor effect, as compared to groups treated with PBS (p = 0.026), INF-α (p = 0.03), A2C9-1 (p = 0.014), PBMC (p = 0.022) or IFN-α+PBMCs (p = 0.007). In fact, the tumor disappeared in 2 of the 4 animals tested. During the course of the experiments we detected no cytotoxicity against normal hepatocytes (data not shown). Histological analysis revealed marked infiltration by mononuclear cells of the residual tumor tissues from mice treated with IFN-α+A2C9-1+PBMCs, but no such infiltration was observed in tumor tissues from mice in the other groups (Figure S3).

### IFN enhances accumulation of anti-FGFR1 mAb within tumors

**Figure 5:**
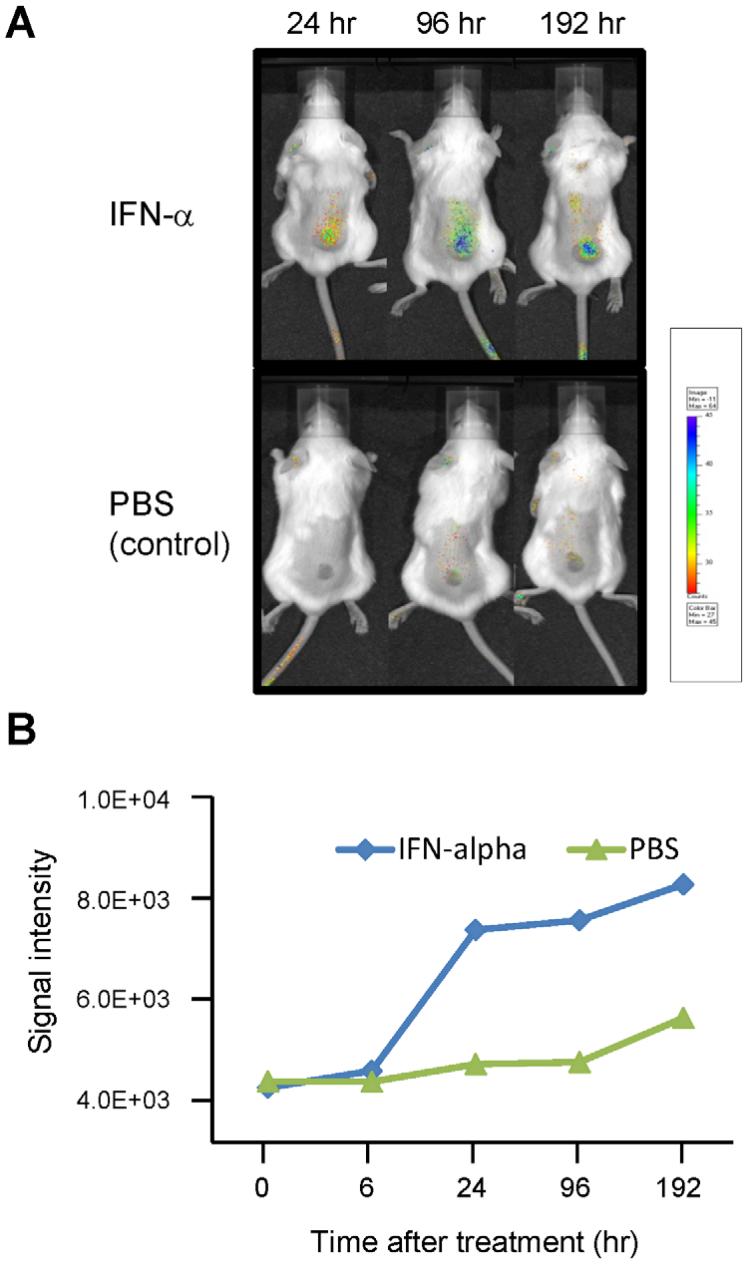
Accumulation of anti-FGFR1 mAb in tumor xenografts is enhanced by IFN-α. A, SCID mice were xenografted with 1×10^6^ HepG2 cells, after which 50 µg of Cy5-conjugated A2C9-1 mAb was intravenously administrated via the tail vein. Mice were then imaged under anesthesia. B, Time course of the Cy5 signal intensity.

To further confirm that the observed regression of the xenografts was related to the treatment with A2C9-1 mAb, Alexa Fluor 680-conjugated A2C9-1 was injected into the tail veins of tumor-bearing SCID mice, after which the targeting of the tumor by A2C9-1 was evaluated in the same animals at different time points using an optical molecular imaging system (Figure 5A and B). In mice pretreated with IFN-α, A2C9-1 selectively and time-dependently accumulated within the tumors during the period from 24 h to 192 h after its administration. By contrast, only negligible levels of mAb were detected in control mice administered A2C9-1+PBS. We also confirmed that there was no accumulation of an Alexa Fluor 680-conjugated control antibody (data not shown). It thus appears that IFN-α enhances the accumulation of anti-FGFR1 mAb *in vivo*, most likely by up-regulating FGFR1.

## Discussion

In this study, we found that FGFR1 can serve as a novel target for antibody therapy in HCC. More specifically, combined treatment with IFN-α/β and an anti-FGFR1 mAb (A2C9-1) showed strong growth suppressive effects on human HCC cells *in vitro* and *in vivo*. Five isoforms of the transmembrane receptor FGFR (FGFR1–4 and FGFR5/1L) are known to be expressed in mammals [12]. Each consists of three extracellular immunoglobulin-like domains, a transmembrane domain, and two intracellular tyrosine kinase domains. FGF binds to the FGFR via two of the immunoglobulin-like domains (II and III). During FGFR expression, alternative splicing of *FGFR* transcripts produces multiple splice variants with different tissue-specific ligand specificities [13]. Among them, FGFR1 has been shown to be expressed in HCC and is known to promote the development of HCC in response to carcinogenic stimulation [14]. FGFR1 is not expressed in noncancerous hepatocytes. FGFR1-mediated signaling is involved in cancer cell growth and infiltration, as well as in angiogenesis [15], which is already a target for antitumor therapies [16]. In addition, previous studies have shown elevated expression of FGFR ligands, including FGF1 and FGF2, in primary HCC tissues and hepatic cancer cell lines [17], [18], [19], [20], strongly suggesting FGF signaling plays a key role in the development of HCC. These characteristics make FGFR1 an attractive molecular target for treating HCC.

One major problem with antibody therapy against cancer is the weak and heterogeneous expression of cell surface antigens. To overcome this problem, we examined genes up-regulated by IFN in HCC xenografts. We found that expression of FGFR1 is induced by IFN-α/β and that treating HCC cells with a combination of IFN-α/β and an anti-FGFR1 mAb effectively inhibits the growth and survival of HCC cells. Thus, one reason for the insufficient therapeutic effect of anticancer drugs targeting FGFR1 appears to be is that, without induction, expression of FGFR1 on cancer cells is not sufficient for effective treatment. Consistent with this idea, our immunohistochemical analysis showed expression of FGFR1 to be very low in untreated HCC cells. Notably, epidermal growth factor receptor (EGFR) is also up-regulated by IFN [21], and this up-regulation of EGFR is a crucial factor underlying the susceptibility of affected cancer cells to anti-EGFR antibody therapy [22]. Taken together, these findings suggest treatment with a combination of IFN and an antibody may be an effective therapeutic strategy against various types of cancer.

The molecular mechanism by which IFN-α/β induces FGFR1 expression remains unknown. It is known, however, that the antitumor and antiviral effects of IFN involve changes in the transcriptional regulation of various genes [23], and that IFN-inducible genes contain an interferon response element (ISRE) in their promoter regions [24]. By using a transcription factor search program, we identified several putative ISREs in the 5′ UTR of FGFR1, suggesting that FGFR1 could be a direct target of type I IFN (data not shown). Further study will be necessary to determine precisely how interferon induces FGFR1.

We also do not yet fully understand the molecular mechanism by which our antibody exerted its anti-tumor effect, though there are several possibilities. Many of the tumor-expressed targets of therapeutic antibodies are growth factor receptors. For example, anti-EGFR antibodies, including Cetuximab, have been shown to block growth factor signaling by preventing the ligand from binding to its receptor, or by preventing receptor dimerization [25]. It is highly likely that A2C9-1 suppresses tumor cell growth through a similar mechanism by targeting IFN-induced FGFR1. It was also reported that the binding of an antibody to a growth factor receptor results in the internalization of the antibody-receptor complex, and the down-regulation of downstream signaling [26]; however, we observed no A2C9-1-induced internalization in cancer cells (data not shown). Thirdly, antibodies against growth factor receptors also exert growth suppressing effects via the immune system [27]. Here, for example, we showed that IFN-α/β enhances the surface expression of FGFR1, perhaps enabling an anticancer effect based on antibody-dependent cell-mediated cytotoxicity to accompany the binding of anti-FGFR1 mAb to the receptor. The results of our *in vivo* experiment showing the importance of PBMCs to the antitumor effects of A2C9-1 is consistent with the idea that this antibody strongly stimulates antibody-dependent cell-mediated cytotoxicity.

In summary, we found that IFN-α/β induces expression of FGFR1 and that treatment with a combination of IFN-α/β and an anti-FGFR1 mAb suppresses HCC cell growth *in vitro* and *in vivo*. We also confirmed that IFN-α/β enhances the accumulation of the anti-FGFR1 mAb within tumors. This treatment protocol selectively inhibits the growth of HCC cells without affecting normal cells, which suggests it could be used in the treatment of HCC without reducing hepatic preliminary performance. We therefore suggest that our results may provide the basis for a novel approach to the treatment of HCC, for which there is no effective therapy at the moment.

## Materials and Methods

### Cell lines and experimental animals

Human hepatic cancer cell lines (HepG2, Huh-7 and CHC4) were obtained from the Japanese Collection of Research Bioresources (Tokyo, Japan) and cultured as recommended. Cells were maintained in Dulbecco's modified Eagle's medium supplemented with 10% fetal bovine serum and penicillin/streptomycin at 37°C under an atmosphere of humidified air with 5% CO_2_. Peripheral blood mononuclear cells (PBMCs) were isolated from healthy volunteers using Ficoll-Paque (GE Healthcare Life Science, Uppsala, Sweden) and used as effector cells in SCID mice. All donors provided written informed consent before collection in accordance with the Declaration of Helsinki, and all protocols using human samples were approved by the institutional review board of Sapporo Medical University. Whole PBMCs (1×10^7^) suspended in 0.2 ml of RPMI 1640 were intraperitoneally injected into each SCID mouse. All animal experiments were conducted in accordance with accepted standards of animal care and approved by the Institutional Animal Care and Use Committee of Sapporo Medical University.

### Microarray analysis

For microarray analysis, HepG2 cells (1×10^6^ cells) were initially xenografted into severe combined immunodeficient (SCID) mice. Three weeks later, when the resultant tumor had reached 6–7 mm in diameter, IFN-α (OIF®; Otsuka Pharmaceutical Co., Ltd., Tokushima, Japan) was subcutaneously injected at a dose of 2000 U/mouse. Samples of tumor tissue were collected before and 24 h after injection of the IFN-α. RNA was extracted from the collected tissues using Trizol (Invitrogen, Carlsbad, CA, USA) and reverse transcribed to cDNA using Superscript III (Invitrogen). The cDNA was then reacted using Gene Navigator cDNA Array Filter-human cancer (Toyobo, Osaka, Japan) and subjected to DNA array analysis using a Fluor-S Multi Imager (Bio-Rad Laboratories, Hercules, CA, USA).

### Real-time RT-PCR analysis

HepG2 (1×10^6^ cells) cells were subcutaneously xenografted into the backs of SCID mice. When the inoculated tumor reached 10 mm in diameter, IFN-α (OIF®) or IFN-β (Feron®; manufactured by Toray Industries, Inc., Tokyo, Japan) was administered intravenously at a dose of 2000 U/mouse, and samples of tumor tissue were collected 0, 1, 3, 8, 24 and 48 h after administration. Total RNA was purified from the samples using a RNeasy Kit (QIAGEN, Hilden, Germany), and single-strand cDNA was synthesized from 1 µg of total RNA using a First-Strand cDNA Synthesis Kit (GE Healthcare Life Science, Uppsala, Sweden). Real-time quantitative analysis was performed using SYBR Green I (Roche, Basel, Switzerland) with a LightCycler Real-time PCR system (Roche). Levels of FGFR1 and OAS1 (control) mRNA expression were normalized to the expression of GAPDH mRNA. Primer sets for FGFR1 (sense, 5′-GGA CGA TGT GCA GAG CAT CAA CTG-3′; anti-sense, 5′-AAC TTC ACT GTC TTG GCA GCC GG-3′), OAS1 (sense, 5′-CAT CCG CCT AGT CAA GCA CTG-3′; anti-sense, 5′-CCA CCA CCC AAG TTT CCT GTA-3′) and GAPDH (sense, 5′-GAA GGT GAA GGT CGG AGT C-3′; anti-sense, 5′-GAA GAT GGT GAT GGG ATT-TC-3′) were synthesized at Greiner Bio-One (Tokyo, Japan).

### Western blot analysis

HepG2, Huh-7 or CHC4 cells were incubated for 48 h in the presence of IFN-α or IFN-β (1,000 IU/ml), after which the cells were lysed in sample buffer (50 mM Tris-HC1, pH 6.8, 6% 2-mercaptoethanol, 2% SDS, 0.004% bromophenol blue, and 10% glycerol). Proteins in samples of lysate were separated by 7.5% polyacrylamide gel electrophoresis and then transferred onto a nitrocellulose membrane (Bio-Rad Laboratories). After blocking the membrane with 2% bovine serum albumin (BSA) in PBS for 1 h at room temperature, it was incubated with anti-human FGFR1 antibody (sc-121, Santa Cruz Biotechnology, Santa Cruz, CA, USA) for 1 h at room temperature. The blot was then developed with 0.005% H_2_0_2_-3, 3′-diaminobenzidine using an immunoperoxidase ABC kit (Vectastain ABC kit, Vector Labs, Burlingame, CA, USA).

### Flow cytometric analysis

Forty-eight hours after administration of IFN-α, expression of FGFR1 was assessed using flow cytometry. Cells in suspension (4×10^5^ cells/tube) were washed with 2 mL of washing buffer (0.2% bovine serum albumin, 0.1% NaN_3_/10 mmol/L phosphate-buffered saline, pH 7.4) and centrifuged at 300 *g* for 5 min at 4°C, after which the supernatant was removed. The remaining cell pellet was fixed in 0.25% paraformaldehyde for at least 15 min in the dark at room temperature, washed twice with 2 mL washing buffer, incubated for 1 h in 70% methanol at 4°C, and then washed again. To examine expression of FGFR1, the fixed cells were incubated first with anti-FGFR1 antibody (1∶100 dilution) for 1 h at 4°C. The cells were then washed twice and incubated for 30 min in 4 mL of fluorescein isothiocyanate-conjugated goat anti-mouse IgG on ice in the dark. After again washing the cells twice, they were suspended in 1 mL of washing buffer for analysis using a FACScan (Becton Dickinson Immunocytometry System, San Jose, CA, USA).

### Analysis of FGFR1 expression in human hepatic cancer xenografts

HepG2 cells (1×10^6^ cells) were xenografted into SCID mice. When the resultant tumor reached 10 mm in diameter, IFN-α (OIF®) or IFN-β (Feron®) was administered intraperitoneally or intravenously at a dose of 100 U/g, and 24 h later tumor tissues were collected. Western blot and immunohistochemical analyses were then performed using an anti-human FGFR1 antibody (sc-121; Santa Cruz Biotechnology).

### Preparation of Anti-FGFR1 Antibody

A polynucleotide encoding the region extending from amino acid 1 to 822 of FGFR1 and represented by SEQ ID NO. 1 was amplified by PCR using full-length FGFR1 (Accession No. NM_000604) as a template with primers No. 5′-3 [(SEQ ID NO. 3): 5′-ACGGGATCCAGGACCCTGGCTGGAGAGACA-3′] and No. 3′-3 [(SEQ ID NO. 4): 5′-AAGCTCGAGCCGCCGGAACCGCGGCCGGA-3′]. The amplified polynucleotide was inserted into pcDNA3.1 (Invitrogen, Carlsbad, CA, USA) to construct an expression vector that was administered as the immunizing antigen at a dose of 50 µg/mouse in a 50-µL volume at 1- or 2-week intervals. The antigen for the initial immunization was admixed with complete Freund's adjuvant, while the antigens for the second and subsequent administrations were admixed with incomplete Freund's adjuvant. Spleen monocytic cells from the immunized mouse and a fusion partner, X63-Ag8-653, were fused using polyethylene glycol-mediated cell fusion, which was followed by selection of a hybridoma using the method of Kinebuchi et al. [28]. Cells that had reacted with the immobilized FGFR1 were cultured in serum-free GIT medium (Wako Pure Chemical Industries, Ltd., Osaka, Japan) to produce mAbs until 80% of the cells had died. The cells were then removed from this medium by centrifugation (1,000 rpm, 15 min), and ammonium sulfate was added to 50% saturation and left overnight at 4°C. The resultant precipitates were recovered by centrifugation (1,000 rpm, 30 min) and dissolved in two-fold diluted binding buffer (Protein AMAPS II kit), after which the IgG was adsorbed onto a protein A column (GE Healthcare Life Science). After eluting the mAbs from the column, the eluate was dialyzed against PBS overnight to purify the antibodies, which yielded a number of mAbs recognizing FGFR1. One of those mAbs was designated A2C9-1 and was confirmed to recognize FGFR1 by Western blotting and FACS using samples of FGFR1 expressed in NIH3T3 cells.

### Affinity measurement

The affinity of anti-FGFR1 mAbs for FGFR1 was determined based on surface plasmon resonance (SPR) using a Biacore 3000 device (Biacore AB, Uppsala, Sweden). The extracellular domain of FGFR1 was covalently coupled to a CM-5 sensor chip at low density (215 response units of FGFR1). Binding kinetics were then assessed using twofold serial dilutions of antibody at concentrations ranging from 500 to 0.08 nM in running buffer (PBS, pH 7.4, 0.005% (v/v), polysorbate 20 – filtered and degassed) at 25°C and a flow rate of 25 ml/min. The regeneration procedure consisted of three injections of 10 ml of 2.5 M guanidinium hydrochloride, after which the sensor chip was flushed for 5 min with running buffer. Statistics and data processing were performed using BIA evaluation software 4.0.1 and GraphPad Prism 4.02 (GraphPad Software Inc., San Diego, CA, USA). All SPR experiments were carried out at Biaffin GmbH & Co. KG (Kassel, Germany).

### Assessing the effect of anti-FGFR1 mAb on hepatic cancer cell viability

To examine the effect of administering IFN-α (OIF®) in combination with anti-FGFR1 mAb (A2C9-1 or A2D11-1) to HCC cells, we assessed the survival rate of HepG2 cells in the presence and absence of IFN-α and/or anti-FGFR1 mAb. HepG2 cells were seeded into the wells of a 24-well plate to a density of 1×10^4^ cells/well, after which anti-FGFR1 mAb, IFN-α or anti-FGFR1 mAb+IFN-α was added, and the culture was continued for 0 to 6 days. The cells were then detached using trypsin, and the survival rate was assessed using MTT assays. Antibody-free culture medium was added as negative control, and cells to which nothing was added were used as an additional control.

### Therapeutic experiment with human hepatic cancer cells-xenografted mouse

HepG2 cells (5×10^6^ cells/mouse) were xenografted subcutaneously into the backs of CB17-scid/scid mice. When the volumes of the tumors reached 100 mm^3^, the mice were divided into 7 treatment groups: 1) Mice in the PBS group received intravenous injections of PBS (250 µL) and normal mouse IgG (100 µg). 2) The IFN-α group received intraperitoneal injections of IFN-α (OIF®: 2000 U) and normal mouse IgG (100 µg). 3) The antibody group received intravenous injections of PBS and anti-FGFR1 mAb (100 µg; A2C9-1). 4) The IFN-α+antibody group received intraperitoneal injections of IFN-α (2000 U) and intravenous injections of anti-FGFR1 mAb (100 µg). 5) The IFN-α+antibody+PBMC group received intraperitoneal injections of IFN-α (2000 U), intravenous injections of anti-FGFR1 mAb (100 µg) and intravenous administration of PBMCs (1×10^7^ cells). 6) The IFN-α+PBMC group received intraperitoneal injections of IFN-α (2000 U) and intravenous administration of PBMCs (1×10^7^ cells). 7) The PBMC group received intravenous administration of PBMCs (1×10^7^ cells). Treatments were administered 5 times in total, beginning on day 0 and then 1 week later (w1), 2 weeks later (w2), 5 weeks later (w5) and 6 weeks later (w6). Only at w6, the antibody dose was increased to 200 µg/mouse. Each group contained 4 animals, and the size of tumor was measured as (major axis)×(minor axis)×2 weekly after initial administration. Tumors were harvested 1 week after the final treatment.

### Immunophotodetection in tumor-bearing mice

HepG2 human HCC cells (1×10^6^ cells) were xenografted into the backs of SCID mice. Three weeks later, when the inoculated tumor had reached about 10 mm in diameter, IFN-α (OIF; Otsuka Pharmaceutical Co., Ltd.) at a dose of 20,000 U/mouse or PBS (control) was intraperitoneally administered. After 24 h, 50 µg of Alexa Fluor 680-conjugated anti-FGFR1 mAb was intravenously administrated via the tail vein. The mice were then imaged under anesthesia using an IVIS LUMINA imaging system (Caliper Life Sciences, Hopkinton, MA, USA).

## Supporting Information

Figure S1 **Induction of** ***FGFR1*** **transcripts by IFN-α and IFN-β.** HepG2 cells (1×10^6^ cells) were subcutaneously xenografted into the backs of SCID mice. When the inoculated tumor had reached 10 mm in diameter, IFN-α or IFN-β was administered intraperitoneally or intravenously at a dose of 2000 U/mouse. Tumor tissues were then collected 0, 1, 3, 8, 24 and 48 h after administration. A, Time-course of FGFR1 and OAS1 (control) mRNA expression following administration of IFN-α. FGFR1 mRNA (blue line) was increased 3 h (151%), 8 h (202%) and 24 h (119%) after administration. OAS1 mRNA (red line) was increased 3 h (162%), 8 h (133%) and 24 h (150%) after administration. Shown are means of two replicates of the real-time RT-PCR. B, Time-course of FGFR1 and OAS1 mRNA expression after administration of IFN-β. FGFR1 mRNA (blue line) was increased 8 h (348%) and 24 h (337%) after administration, while OAS1 mRNA (red line) was increased 3 h (262%) and 8 h (511%) after administration. The levels of mRNA expression were normalized to that of GAPDH mRNA. The expression level at 0 h was taken as 100%.
(TIFF)

Figure S2 **Evaluation of anti-FGFR1 monoclonal antibodies.** A, Western blot analysis for FGFR1 in NIH3T3 cells stably transfected for FGFR1. The antibodies used are shown below the panel. B, Surface plasmon resonance analysis. The affinity of anti-FGFR1 mAb for FGFR1 was determined based on surface plasmon resonance. The extracellular domain of FGFR1, which was fused to the constant region of mouse IgG1, was covalently coupled to a CM-5 sensor chip at a density of 3400 response units. Binding kinetics were determined using two-fold serial dilutions of antibody at concentrations ranging from 200 to 12.5 nM in running buffer (PBS, pH 7.4, filtered and degassed). The regeneration procedure was carried using 15 µL of 3 M sodium thiocyanate. B, The apparent association and dissociation rate constants (ka1 (1/Ms) and kd1 (1/s)) and Kd values for A2C9-1 and A2D11-1.
(TIFF)

Figure S3 **Histological analysis of human hepatic cancer cell-xenograft tumors.** Hematoxylin and eosin (HE) staining of xenograft tumors from mice treated with PBMC only, IFN-α only, A2C9-1 only, IFN-α+A2C9-1 and IFN-α+A2C9-1+PBMC. Tumors were harvested 1 week after the final treatment. Note the marked infiltration by mononuclear lymphocytes of tumors from mice treated with IFN-α+A2C9-1+PBMC and the absence of infiltration of tumors from the other groups.
(TIFF)
