# Supplementary figures and images for: Interferon-α/β and Anti-Fibroblast Growth Factor Receptor 1 Monoclonal Antibody Suppress Hepatic Cancer Cells In Vitro and In Vivo
Source: PLoS One. 2011 May 9;6(5):e19618. doi: 10.1371/journal.pone.0019618 (PMC3090414; doi:10.1371/journal.pone.0019618)

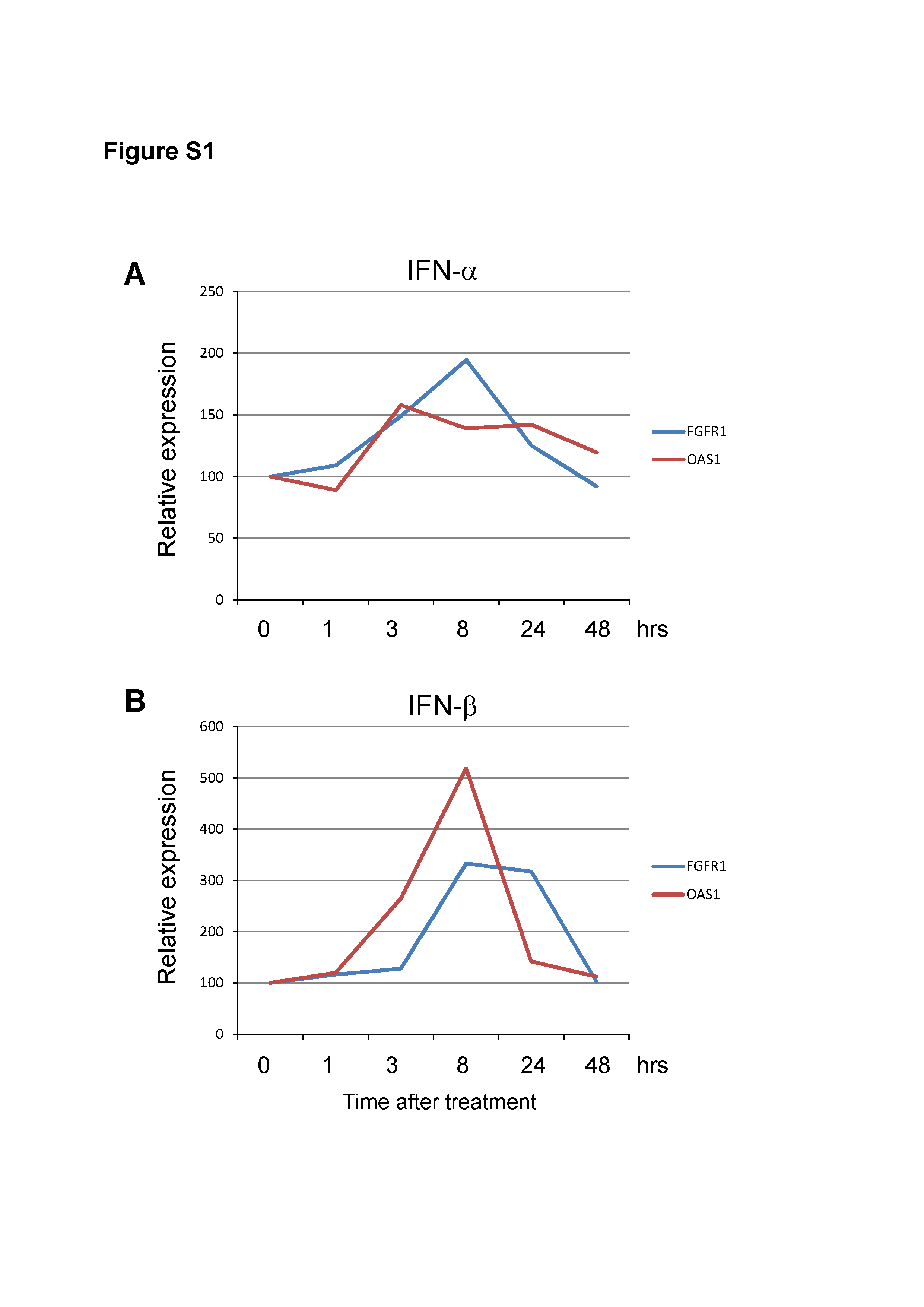

Supplement: Figure S1 — Induction of FGFR1 transcripts by IFN-α and IFN-β. HepG2 cells (1×10^6^ cells) were subcutaneously xenografted into the backs of SCID mice. When the inoculated tumor had reached 10 mm in diameter, IFN-α or IFN-β was administered intraperitoneally or intravenously at a dose of 2000 U/mouse. Tumor tissues were then collected 0, 1, 3, 8, 24 and 48 h after administration. A, Time-course of FGFR1 and OAS1 (control) mRNA expression following administration of IFN-α. FGFR1 mRNA (blue line) was increased 3 h (151%), 8 h (202%) and 24 h (119%) after administration. OAS1 mRNA (red line) was increased 3 h (162%), 8 h (133%) and 24 h (150%) after administration. Shown are means of two replicates of the real-time RT-PCR. B, Time-course of FGFR1 and OAS1 mRNA expression after administration of IFN-β. FGFR1 mRNA (blue line) was increased 8 h (348%) and 24 h (337%) after administration, while OAS1 mRNA (red line) was increased 3 h (262%) and 8 h (511%) after administration. The levels of mRNA expression were normalized to that of GAPDH mRNA. The expression level at 0 h was taken as 100%. (TIFF) [file pone.0019618.s001.tif]

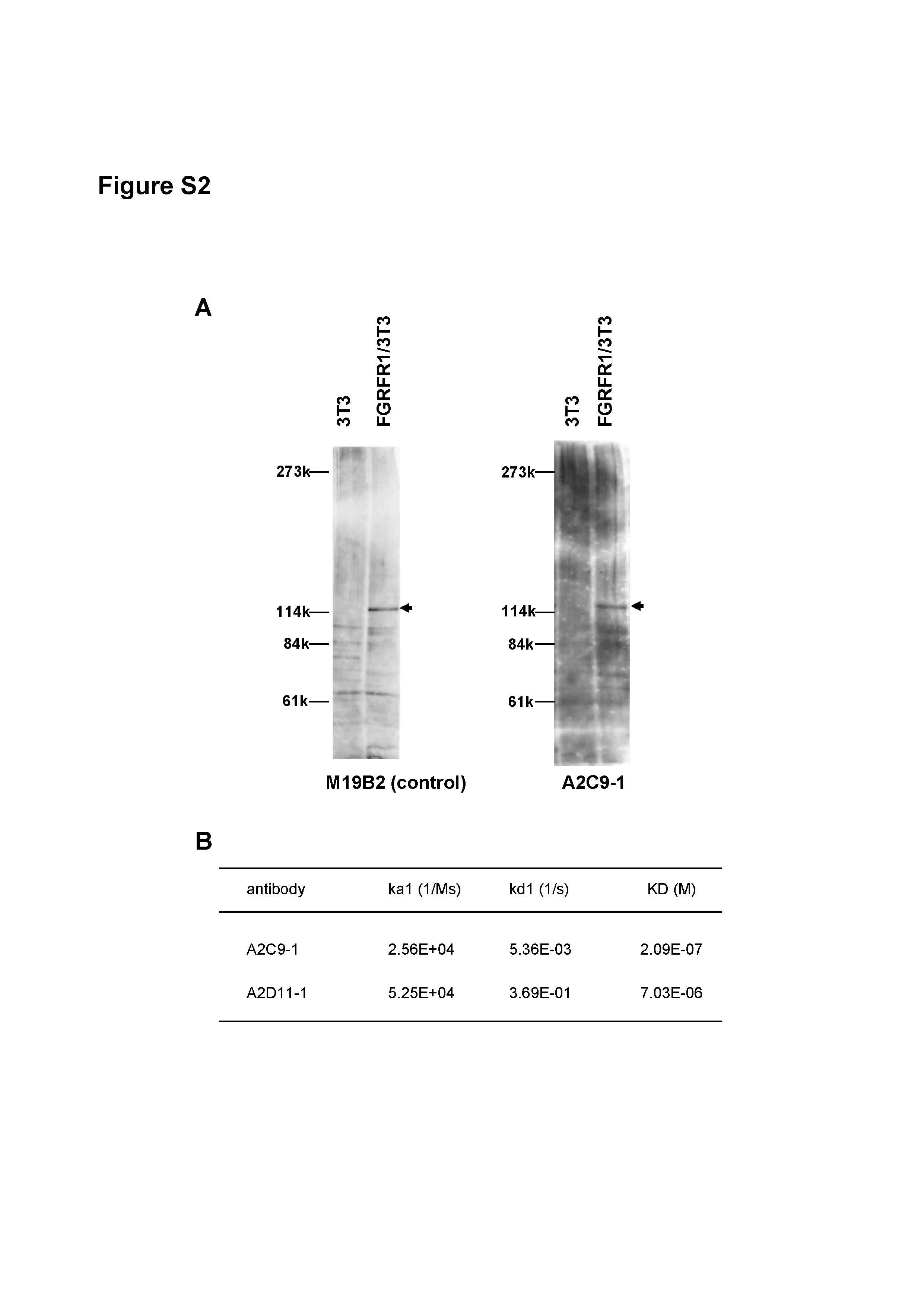

Supplement: Figure S2 — Evaluation of anti-FGFR1 monoclonal antibodies. A, Western blot analysis for FGFR1 in NIH3T3 cells stably transfected for FGFR1. The antibodies used are shown below the panel. B, Surface plasmon resonance analysis. The affinity of anti-FGFR1 mAb for FGFR1 was determined based on surface plasmon resonance. The extracellular domain of FGFR1, which was fused to the constant region of mouse IgG1, was covalently coupled to a CM-5 sensor chip at a density of 3400 response units. Binding kinetics were determined using two-fold serial dilutions of antibody at concentrations ranging from 200 to 12.5 nM in running buffer (PBS, pH 7.4, filtered and degassed). The regeneration procedure was carried using 15 µL of 3 M sodium thiocyanate. B, The apparent association and dissociation rate constants (ka1 (1/Ms) and kd1 (1/s)) and Kd values for A2C9-1 and A2D11-1. (TIFF) [file pone.0019618.s002.tif]

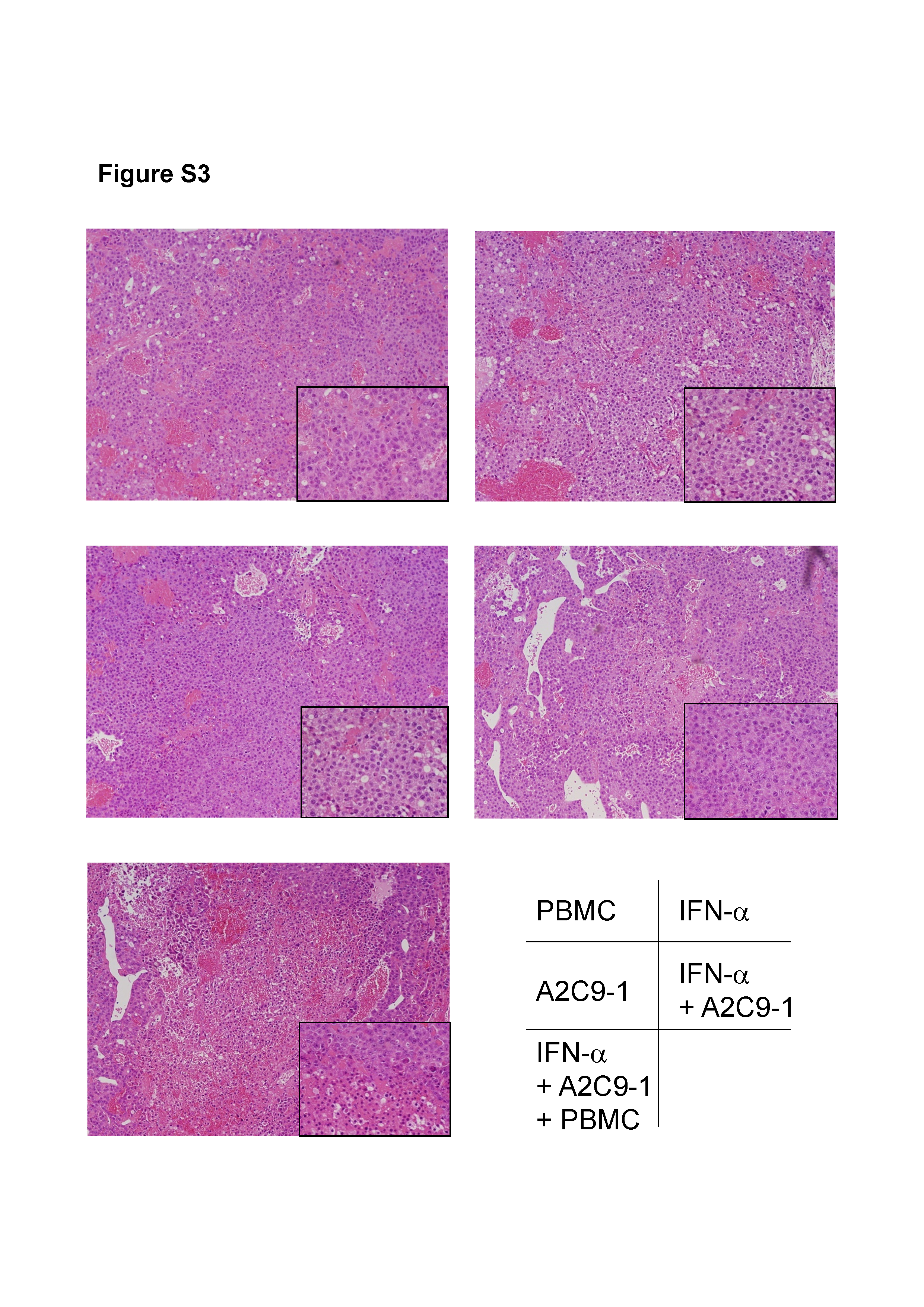

Supplement: Figure S3 — Histological analysis of human hepatic cancer cell-xenograft tumors. Hematoxylin and eosin (HE) staining of xenograft tumors from mice treated with PBMC only, IFN-α only, A2C9-1 only, IFN-α+A2C9-1 and IFN-α+A2C9-1+PBMC. Tumors were harvested 1 week after the final treatment. Note the marked infiltration by mononuclear lymphocytes of tumors from mice treated with IFN-α+A2C9-1+PBMC and the absence of infiltration of tumors from the other groups. (TIFF) [file pone.0019618.s003.tif]
